# A Method for Estimating the Number of Infections From the Reported Number of Deaths

**DOI:** 10.3389/fpubh.2021.648545

**Published:** 2022-01-20

**Authors:** Åke Brännström, Henrik Sjödin, Joacim Rocklöv

**Affiliations:** ^1^Department of Mathematics and Mathematical Statistics, Umeå University, Umeå, Sweden; ^2^Advancing Systems Analysis Program, International Institute for Applied Systems Analysis, Laxenburg, Austria; ^3^Department of Public Health and Clinical Medicine, Section of Sustainable Health, Umeå University, Umeå, Sweden

**Keywords:** COVID-19, infectives, estimating, nowcasting, surveillance

## Abstract

At the outset of an epidemic, available case data typically underestimate the total number of infections due to insufficient testing, potentially hampering public responses. Here, we present a method for statistically estimating the true number of cases with confidence intervals from the reported number of deaths and estimates of the infection fatality ratio; assuming that the time from infection to death follows a known distribution. While the method is applicable to any epidemic with a significant mortality rate, we exemplify the method by applying it to COVID-19. Our findings indicate that the number of unreported COVID-19 infections in March 2020 was likely to be at least one order of magnitude higher than the reported cases, with the degree of underestimation among the countries considered being particularly high in the United Kingdom.

## Introduction

In the spring of 2020, the world experienced the outbreak of the novel corona virus SARS-CoV-2 which rapidly spread across the globe and was declared a pandemic by the World Health Organization on March 11 (1). While China initially appeared successful in containing the outbreak, the virus had by January 20 caused an outbreak on a cruise ship outside the coast of Japan (2), and by January 31 it had also spread to northern Italy (3) and further on to practically all countries in Europe, as well as other countries across the globe. In the United States of America, the outbreak seemed at first under control, yet, it soon become clear that the transmission had grown out of control in several regions, in particular in New York. At the time of writing (July 26, 2021), the virus has likely spread to all countries globally, with more than 400.000 reported deaths due to COVID-19.

With limited testing capacity and few statistical sampling studies early in the pandemic, the true number of infected individuals initially remained unknown. This is a general problem with any novel pathogen, as it takes time to establish testing procedures for virus and antibodies and carry out epidemiological samples and statistical analyses. At an early phase, statistical testing is especially difficult due to the need for large samples to reduce uncertainty in estimates. Having access to reliable estimates for the true number of infections will be important for risk assessments and determining effective strategies, and is also of public interest. With the lack of timely access to representative samples, there is accordingly a need for effective methods to estimate the number of infections during an epidemic, especially for translating internationally emerging knowledge to local contexts where such data is missing.

Here, we address this problem by showing how to estimate the true number of infections in an epidemic from the reported number of deaths. The estimation procedure requires knowledge of the distribution of times from infection to death and the infection fatality ratio (i.e., the proportion of infected individuals that dies from the infection). We exemplify our method by estimating the true number of infections in the early spread of COVID-19 for selected countries and find that the true number of infections significantly exceeded the reported number of infections at that time. The difference can be up to several orders of magnitude depending on the location.

## Statistical Method

We consider an epidemic with an infection fatality ratio that is sufficiently high for the outbreak to be detected and deaths reported, say in excess of 0.1 %. For simplicity, we first describe our method under the specific assumption that infections grow exponentially, i.e., with a fixed doubling time. This will typically be the case early in an epidemic, until either mitigating measures have been put in place or the number of susceptible individuals has been significantly depleted (4). We then present a more general case in which an arbitrary growth model, such as the generalized Richards model, is fitted to reported fatality data. We assume knowledge of the distribution of time from infection to death and of the infection fatality ratio. In all estimates of the number of infected, we include both dead and recovered individuals, i.e., we estimate the cumulative number of infections.

### Heurestic Estimate Assuming Exponential Growth

**Figure 1:**
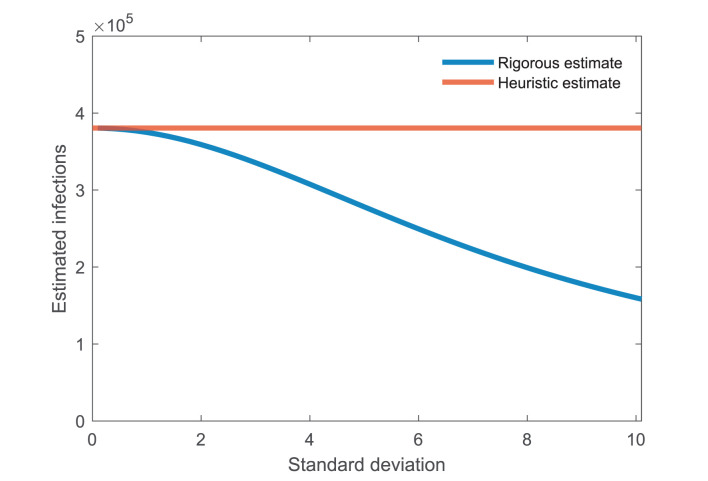
Not accounting for the standard deviation in time can lead to large estimation error. The blue curve shows the estimated number of infectives assuming that the time from infection to death is lognormally distributed with mean 21 days and standard deviation given by the horizontal axis. The heuristic estimate using Equation 1, shown as the red curve, underestimates the actual number up to four times. Other parameters: exponential doubling time *T* = 4, number of deaths *d* = 100, and infection fatality ratio *p* = 0.01 (i.e., 1%).

Initially, before formalizing the method, we consider a simpler case when individuals die exactly *S* days after being infected with a probability equal to the infection fatality ratio. For this simpler case, we can estimate the number of infected individuals *S* days ago by dividing the reported number of deaths with the infection fatality ratio. Assuming exponential growth with a doubling time of *T* days, the number of infected individuals at the present day can then be estimated as


(1) $$\begin{array}{r} N=\frac{d}{p\;}\exp\left(S\frac{\;\ln\;2}{T}\right), \end{array}$$

where *d* is the number of deaths, *p* is the infection fatality ratio, and *T* is the exponential doubling time. For example, if the doubling time is *S*/2 we would estimate four times as many infected individuals today, as the number of infected individuals doubled twice before having an impact on the number of reported deaths. We will extend this reasoning by assuming a cumulative probability function Θ(*t*) u of the time from infection to death, such the probability that an individual who was infected t days ago is dead on the present day is *p*Θ(*t*), where *p* is the infection fatality ratio. It may be tempting to heuristically estimate the number of infections using Equation 1 with *S*u as the average time from infection to death, but as we show in Figure 1, this leads to a large estimation error when there is large variability in the time from infection to death, even when the assumption of exponential growth in the number of infections is correct. Hence, a more rigorous approach is called for.

### Rigorous Estimate Assuming Exponential Growth

We write *N* for the number of infected individuals on the present day. Under our assumption of exponential growth in the number of infections with doubling time *T*, the number of infected individuals *t* days ago is $N\exp\left(-\frac{t\ln\;2}{T}\right)$. The inflow of new infectives *t* days ago^1^ can then be found by differentiating, giving $\ln\;\left(2\right)\frac{N}{T}\exp\left(-\frac{t\ln\;2}{T}\right)$. The number of deaths on the present day, *d*, can then be written as


$$\begin{array}{l} d=\;\int_{0}^{\infty }\left(\ln\;2\right)\frac{N}{T}\exp\left(-\frac{t\ln\;2}{T}\right)p\Theta \left(t\right)\text{ dt}. \end{array}$$

Let


$$\begin{array}{l} k\left(T\right)=\;\int_{0}^{\infty }\left(\ln\;2\right)\frac{1}{T}\exp\left(-\frac{t\ln\;2}{T}\right)\Theta \left(t\right)\text{ dt}. \end{array}$$

The equation can then be written


$$\begin{array}{l} d=Npk\left(T\right). \end{array}$$

We can thus estimate the number of infectives as


(2) $$\begin{array}{r} N=\frac{d}{pk\left(T\right)\;}. \end{array}$$

In the special case in which individuals die exactly after *S* days, i.e., Θ(*t*) = *H*(*t* − *S*) where *H* is the Heaviside or unit step function, we find that $k\left(T\right)=\exp\left(-S\frac{\ln\;2}{T}\right)$ and hence


$$\begin{array}{l} N=\frac{d}{p}\exp\left(S\frac{\ln\;2}{T}\right), \end{array}$$

which is identical to Equation 1. Figure 1 shows how the rigorous estimate, Equation 2, differs from the heuristic estimate, Equation 1. The heuristic estimate consistently overestimates the number of infectives. In the example shown, the error becomes twofold assuming an average of 21 days from death to infection with a standard deviation of 8 days.

### Sensitivity Analysis Assuming Exponential Growth

**Figure 2:**
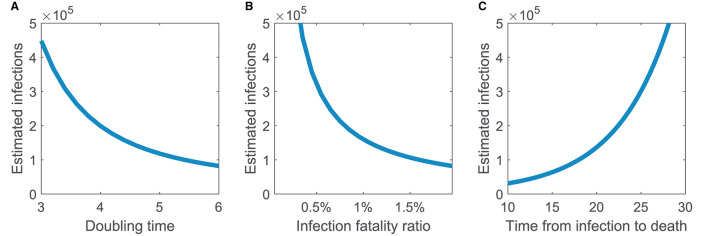
Estimated number of infections assuming exponential growth is robust to small variations in key parameters. How the estimated number of infections depends on **(A)** doubling time, **(B)** infection fatality ratio, and **(C)** average time from infection to death. Parameters not varied are doubling time *T* = 4, number of deaths *d* = 100, and infection fatality ratio *p* = 0.01 (i.e., 1%). Time from infection to death is assumed lognormally distributed with mean 21 days and standard deviation 8 days, except for the right panel where the mean time is given by the horizontal axis.

Figure 2 shows how the estimated number of infectives varies with key parameters: doubling time, infection fatality ratio, and average time from infection to death. The estimate depends non-linearly on all three parameters considered. The estimate scales linearly with the number of deaths (not shown). We conclude that the estimate will be robust for small variations in these key parameters.

### Rigorous Estimate for Other Growth Modes

In the presentation thus far, we have assumed that the cumulative number of infections grow exponentially. This is a reasonable assumption early in an epidemic and has the dual advantages that the only required parameter—the doubling time—can directly be estimated from fatality data and that we obtain a closed formula, Equation 2, for the estimated number of infected. Our method can, however, be used also with other assumptions, including the generalized epidemic growth model, the Richards model, and the generalized logistic model [see, e.g., (5–9)]. Here, we present our method in the case of a general function describing the growth in the cumulative number of infections. In this case, the growth function cannot be directly fitted to the fatality data and there is typically no closed formula for the number of infected. Instead, we estimate the parameters of this function and the cumulative number of infections from a time series of reported deaths.

Assume that the number of infected that can be written on the form *f*(*t, N*, θ) where *N* is the number of infected on the present day, *t* time in days relative to the present day, and θ parameters describing the growth rate. With the same notation as before, we can now determine the cumulative number of deaths *t* days ago as


$$\begin{array}{l} d_{t}=\;-\int_{t}^{\infty }\frac{\partial f}{\partial t}\left(-s,N,\theta \right)\;p\Theta \left(s-t\right)\text{ d}s. \end{array}$$

The parameters θ are inferred by fitting to the reported cumulative number of deaths. Writing $\tilde{d_{t}}$ for these empirical values, estimates of *N* and θ can be found by standard least-square minimization using reported cumulative deaths from the last *n* + 1 days.


$$\begin{array}{l} \left(N,\theta \right)=\text{argmin }\overset{n}{\underset{t=0}{\sum }}{\left(d_{t}-\;\tilde{d_{t}}\right)}^{2}. \end{array}$$

For the special case when *f*(*t, N*, θ) = *Ng*(*t*, θ), the parameters θ are known, and *n* = 1, we only have the term ${\left(d_{0}-\;\tilde{d_{0}}\right)}^{2}$in the sum. This is minimized when $d_{0}=\tilde{d_{0}}$. Solving this equation gives


$$\begin{array}{l} N=\;\frac{\tilde{d_{0}}}{-p\int_{0}^{\infty }\frac{\partial g}{\partial t}\left(-s,\theta \right)\;\Theta \left(s\right)\text{ d}s}. \end{array}$$

Writing θ = *T* and letting $g\left(t,T\right)=\text{ exp}\left(\frac{t\ln\;2}{T}\right)$, we obtain


$$\begin{array}{l} N=\;\frac{\tilde{d_{0}}}{pk\left(T\right)}, \end{array}$$

with *k*(*T*) defined as before. Thus, the estimate we derived in section Rigorous Estimate Assuming Exponential Growth is a special case of least-squares estimation when the doubling time is already known. Note that the least squares estimation can also be done using daily deaths rather than cumulative deaths, and that this is likely preferable in practical applications.

### Confidence Intervals

We use model-based bootstrapping to determine indicative confidence intervals (10, 11). Given the estimate *N* of the number of infected and θ for the parameters of the growth model, we simulate a large number of fatality time series. From each of these, we estimate the number of infected and determine the confidence intervals as the quantiles of these estimates. Specifically, we assume that the number of new cases *t* days ago was *n*_*t*_ = *f*(−*t, N*, θ)−*f*(−*t* − 1, *N*, θ) rounded off to the nearest integer. We classify each of these cases as a fatality with probability *p* equal to the infection fatality ratio and draw the time at which the fatality occurs from the probability distribution Θ. This allows us to determine a time series of simulated fatalities, ${\hat{d}}_{t}$, of individuals who, in the simulation, died on or before day *t*. For each such time series, we determine an associated estimate $\hat{N}$ and by ordering a large number of such estimates from the smallest to the largest, we determine confidence bounds *N*_0.025_ and *N*_0.975_ such that 95% of all simulated estimates $\hat{N}$ lie between these two values.

For the specific case of exponential growth with known doubling time, described in Section Rigorous Estimate Assuming Exponential Growth, the process above can be greatly simplified. As we assume statistical independence between individuals, the number of dead individuals at the present day in any given simulation is approximately normally distributed with mean and variance,


$$\begin{array}{l} \mu =\;\overset{\infty }{\underset{t=1}{\sum }}n_{t}p\Theta \left(t\right), \end{array}$$

$$\begin{array}{l} \sigma^{2}=\overset{\infty }{\underset{t=1}{\sum }}n_{t}p\Theta \left(t\right)\left(1-p\Theta \left(t\right)\right), \end{array}$$

where *pΘ*(*t*) is the probability that an individual infected *t* days ago is now dead. We then determine the 2.5% and 97.5% quantiles, d_0.025_ and d_0.975_ and estimate a 95% confidence interval for the number of infected individuals as *N*_0.025_ = *d*_0.025_/(*pk*(*T*)) to *N*_0.975_ = *d*_0.975_/(*pk*(*T*)). Note that, as the doubling time is assumed known, the confidence intervals determined through this simplified approach will not encompass uncertainty in the underlying increase in infections; it accounts only for the uncertainty in number of deaths resulting from these infections.

## Application to COVID-19

To illustrate the method, we apply it to estimate the number of infected individuals depending on the number of deaths for the early phase of COVID-19 spread. We assumed that the time from infection to onset of symptoms illness onset and the time from illness onset to death are lognormally distributed with an average time of 5.6 and 15.0 days, respectively, and a standard deviation of 3.9 and 6.9 days, respectively [(12); Table 2]. Assuming that these times are statistically independent, we determined the average time from infection to death as 20.6 days with a standard deviation of 7.9 days. Before suppressive measures was fully in place, the number of deaths in Europe increased exponentially with a typically doubling time around 3 days (13). In China, the doubling time was estimated to 7.6 days (14). We assumed a doubling time of 4 days. Finally, we assumed an infectious fatality ratio of 0.8%.

**Figure 3:**
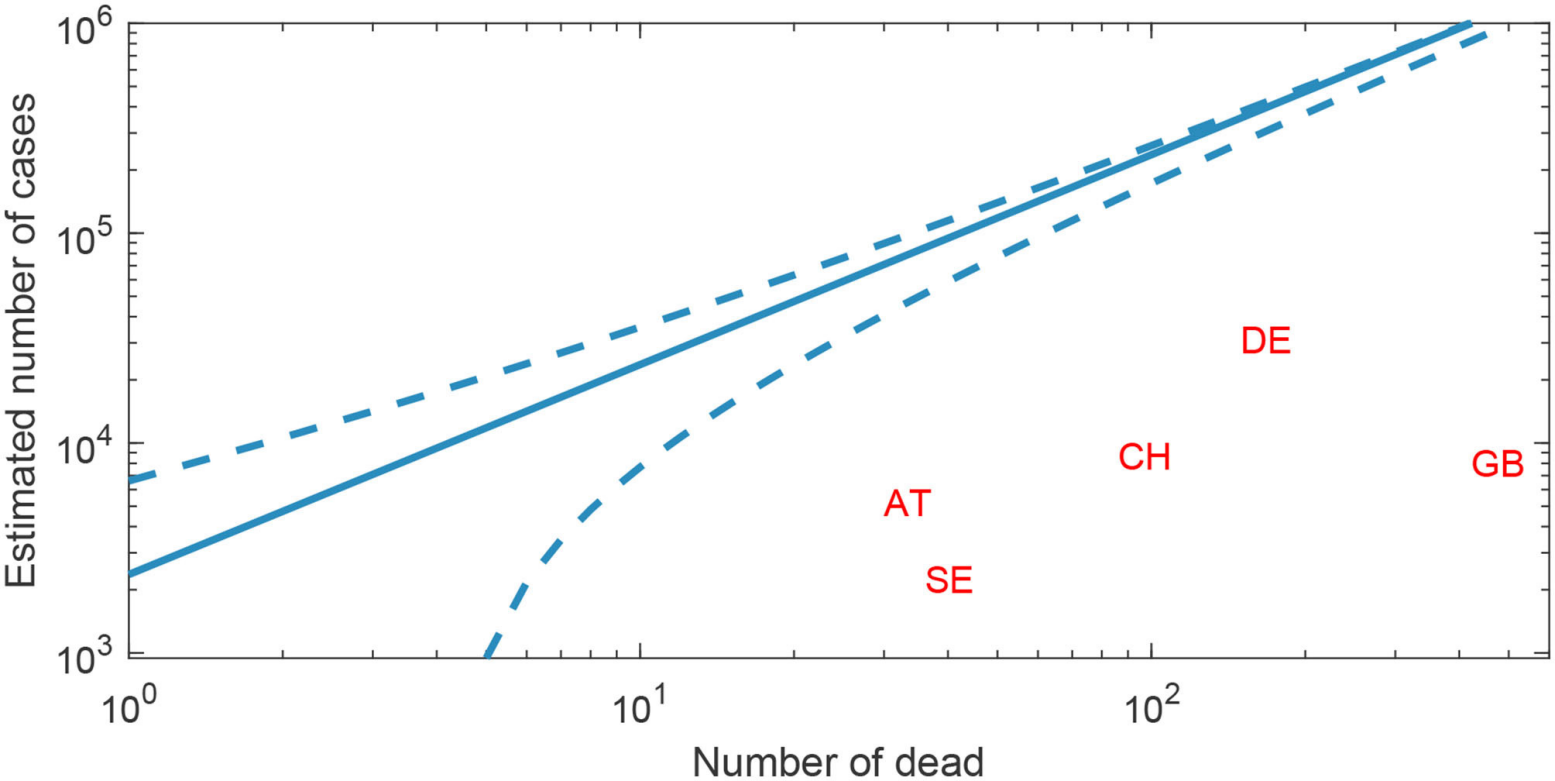
Predicted number of COVID-19 cases based on the reported number of deaths. The predicted cumulative number of COVID-19 cases (solid line) depending on the number of reported deaths, with 95% indicative confidence intervals (dashed line), here based on a doubling time of 4 days and an infectious fatality ratio of 0.8%. Two letter country codes indicate the country's reported deaths and cases based on European Center for Disease Prevention and Control data from March 25.

Figure 3 shows our estimate for the number of individuals infected by COVID-19 on March 25 in five selected countries. In all cases, the estimated number of infectives greatly exceeds the reported number of cases at the time, but there is a large variability between countries. In Germany, the estimated number is 11 times larger than the reported number. By contrast, for Great Britain the estimated number is 123 times larger than the reported number. For Sweden, we estimate the number of infected individuals on March 25 was 85,094 (95% CI 51, 498–104, 640). Note that some of individuals would have recovered by this date, but if infections increased exponentially to this date, this should be a rather small fraction as most individuals would recently have been infected. Slightly different values would be attained if calibrating the infection fatality ratio and the doubling time specifically to respective countries.

Finally, using the method presented in Section Rigorous Estimate for Other Growth Modes, we fit a generalized growth model [see, e.g., (6)] to cumulative COVID-19 fatality data from the Swedish National Board of Health and Welfare for the 12 days up until and including March 29, 2020. The generalized growth model assumes that the number of infected *N* changes according to the differential equation *N*′ = *rN*^*q*^, in which the exponent *q* determine if the growth is sub-exponential (0 < *q* < 1), exponential (*q* = 1), or super-exponential (*q*>1). Using the Levenberg–Marquardt algorithm (15), implemented as part of lsqnonlin in MATLAB R2021a, we estimate parameters *r* = 14.8, *q* = 0.58, and 218 600 infected by March 29 which appears consistent with our estimate for Sweden on March 25 in Figure 3.

## Discussion

Information about the number of infected individuals is important during epidemics to inform policy and of great public interest. Yet, this number is difficult to estimate, especially early in an epidemic when widespread testing may not be available and random sampling has not been carried out. We have shown how the cumulative number of infected individuals may be estimated with confidence intervals for any infectious disease for which the infection fatality rate is significant. Our method is primarily developed and presented under the assumption of a fixed doubling time, which is likely to hold in the early phase of an epidemic, but in section Rigorous Estimate for Other Growth Modes we also show how general growth models can be fitted to time-series data. This increases the applicability of our method to later stages of an epidemic when mitigation measures and/or widespread immunity slows the spread of infections.

In applying our findings to the early spread of COVID-19, we found that the number of reported cases were one or two orders of magnitude less than our estimates. As suggested by one of the reviewers to this manuscript, it would be interesting to apply the methodology to country-specific case fatality ratios rather than a single estimated infection fatality ratio and look for patterns in the fraction of excess cases one would obtain for each country. Having this methodology available in an early stage of the pandemic can provide invaluable help to public health professionals and policy makers to early assessment, and mitigation and suppression of health impacts.

An important finding of our work is that a heuristic estimation of the number of infected individuals based only on the average time from infection to death may, in the early exponential growth phase of an epidemic, greatly overestimate the true number of infected individuals (Figure 1). A similar heuristic estimation is, for example, used in (16) to study disease dynamics in Stockholm. Our intuitive understanding of why this discrepancy occurs is that most infections during exponential growth will have occurred recently, with half having occurred within the exponential doubling time. If the exponential doubling time is shorter than the average time from infection to death and the standard deviation is large, most individuals who have died will have done so recently. Hence, the heuristic method will overestimate the true number of infections as typically most deaths will have occurred much more recently in time.

A limitation of our approach is that we require knowledge of the infection fatality ratio, the distribution of time from infection to death, and the initial growth curve for the number of infections. Despite this, the approach can potentially be very useful for early risk assessment, as globally emerging knowledge can be applied in local settings to estimate infection rates and guide early action to curb an epidemic outbreak. For COVID-19, the distribution of time from infection to death can be estimated using data in Wuhan, China (12), while reasonable but uncertain values for the infection fatality ratio was available from studies of regional and local outbreaks, such as South Korea (17) and the cruise ship the Diamond Princess (2). The assumption of exponential growth with a fixed doubling time was supported by epidemiological theory and by the reported number of deaths early in the epidemic. Country-specific calibration of these parameter values would naturally improve per-country estimate accuracy, as, for instance, the infection fatality ratio normally is dependent on the age-distribution of a population. Another alternative would be to include the statistical dispersion of these parameter in the derivation of indicative confidence intervals. These would then likely show slightly wider interval compared to the ones currently applied in Figure 3 and based only on stochasticity in deaths. Notably, at later stages of an epidemic, the cumulative number of infections cannot be assumed to grow exponentially with a fixed doubling time. In the appendix, we describe a generalization that allows fitting other functions describing the growth in the cumulative number of cases.

While our methodology has limitations and the resulting estimates have uncertainties, we believe that it can offer valuable guidance during an epidemic. Further studies of COVID-19 and other epidemics, including random sampling of populations, will help to elucidate the reliability of our approach. For the time being, we believe this is one of the most efficient and accurate way of obtaining indicative estimates of the number of infections in the early stages of emerging epidemics.

## Data Availability Statement

The original contributions presented in the study are included in the article/supplementary material, further inquiries can be directed to the corresponding author.

## Author Contributions

ÅB, HS, and JR: conceptualization. ÅB: methodology and software. ÅB, HS, and JR: validation. ÅB and HS writing—original draft preparation. JR: writing—review and editing. All authors have read and agreed to the published version of the manuscript.

## Conflict of Interest

The authors declare that the research was conducted in the absence of any commercial or financial relationships that could be construed as a potential conflict of interest.

## Publisher's Note

All claims expressed in this article are solely those of the authors and do not necessarily represent those of their affiliated organizations, or those of the publisher, the editors and the reviewers. Any product that may be evaluated in this article, or claim that may be made by its manufacturer, is not guaranteed or endorsed by the publisher.

## Footnotes

^1^Note that his quantity is necessarily positive. A direct differentiating gives a negative value as *t* represents days into the past.
